# Access to primary health care for asylum seekers and refugees: a qualitative study of service user experiences in the UK

**DOI:** 10.3399/bjgp19X701309

**Published:** 2019-02-12

**Authors:** Cara Kang, Louise Tomkow, Rebecca Farrington

**Affiliations:** School of Biology, Medicine, and Health;; Humanitarian and Conflict Response Institute, School of Arts, Languages, and Cultures, University of Manchester, Manchester.; School of Biology, Medicine, and Health;

**Keywords:** access to health care, asylum seekers, qualitative research, refugees

## Abstract

**Background:**

Asylum seekers and refugees (ASR) face difficulty accessing health care in host countries. In 2017, NHS charges for overseas visitors were extended to include some community care for refused asylum seekers. There is growing concern that this will increase access difficulties, but no recent research has documented the lived experiences of ASR accessing UK primary health care.

**Aim:**

To examine ASR experiences accessing primary health care in the UK in 2018.

**Design and setting:**

This was a qualitative community-based study. ASR were recruited by criterion-based sampling through voluntary community organisations.

**Method:**

A total of 18 ASR completed face-to-face semi-structured recorded interviews discussing primary care access. Transcripts underwent thematic analysis by three researchers using Penchansky and Thomas’s modified theory of access.

**Results:**

The qualitative data show that participants found primary care services difficult to navigate and negotiate. Dominant themes included language barriers and inadequate interpretation services; lack of awareness of the structure and function of the NHS; difficulty meeting the costs of dental care, prescription fees, and transport to appointments; and the perception of discrimination relating to race, religion, and immigration status.

**Conclusion:**

By centralising the voices of ASR and illustrating the negative consequences of poor healthcare access, this article urges consideration of how access to primary care in the UK can be enhanced for often marginalised individuals with complex needs.

## INTRODUCTION

Migrants use emergency health services more, and for lower acuity conditions, than non-migrants across Europe.^1^ It has been speculated that this is due to barriers accessing primary care. Research shows that asylum seekers and refugees (ASR) have diverse and additional healthcare needs to host populations.^2^^–^5 Multiple sources suggest ASR face difficulty accessing appropriate health care in their host countries.^6^^–^10 A recent European literature review highlighted difficulties with communication, poor doctor–patient relationships, and culturally inappropriate primary care.^11^

In 2012, as part of the government’s aim to decrease immigration from the *‘hundreds of thousands to the tens of thousands’*, then Home Secretary Theresa May declared the UK would become a *‘really hostile environment’* for those classified as irregular migrants.^12^ The policy changes that ensued restricted refused asylum seekers’ entitlements to services, including NHS care.^13^^,^^14^ In 2017, charging for health care was extended, with costs to be paid before treatment. Though GP services remain free, charges now apply to community services allied to primary care.^15^ Doctors of the World UK recently found that 13% of vulnerable migrants who attempted to register with a GP were incorrectly refused because of their immigration status.^16^ There is growing concern from both practitioners and migrant-rights advocacy groups that changes to charging regulations amplify access difficulties.^15^^,^^17^^–^20

Healthcare access can be defined as the degree of fit between patients and the healthcare system.^21^ Penchansky and Thomas suggest that access consists of specific, yet overlapping, dimensions — accessibility, availability, acceptability, affordability, and accommodation.^22^ Saurman later expanded this theory to include awareness (Box 1).^21^

**Box 1.** The dimensions of access^a^

**Dimension**	**Definition**	**Components and examples**
Accessibility	Location	Proximity to the patient in time and distance
Availability	Supply and demand	Sufficient services and resources to meet volume and needs of the patient
Accommodation	Organisation	Well organised to accept patients, and patients are able to use the services. This includes hours of operation, appointment systems, and facility structures (for example, wheelchair access)
Affordability	Financial cost	Direct and indirect costs for patients
Acceptability	Consumer perception	The attitude of patient towards the care provider and the characteristics of the service, including social and cultural concerns
Awareness^b^	Communication and information	Effective communication and information strategies with patients, including consideration of context and health literacy

a *Adapted from Patel* et al*, 2017.^16^*

b Modified as per Saurman, 2016.^21^

There have been recent calls for research on country-specific issues surrounding ASR access to primary care.^11^ In the UK, existing research into ASR access to health care was either conducted before the implementation of new charging regulations,^23^^–^25 or focuses specifically on secondary care.^26^ No studies examine refugees’ and asylum seekers’ experiences of accessing primary care since the implementation of new charging regulations. This study addresses this gap, using Penchansky and Thomas’s modified theory of access as its analytical framework.

## METHOD

### Sample recruitment

Criterion-based sampling was used to recruit ASR who had resided in the UK for <5 years and accessed primary health services in the past 2 years.^27^ Fliers outlining the research process and inclusion criteria were distributed to voluntary and community organisations (VCOs) supporting ASR in Greater Manchester. The VCOs run free community drop-ins that provide non-medical support and activities to ASR of all ages and backgrounds. VCO staff facilitated enrolment. Informed consent was obtained through the provision of information in an accessible format. Participants were briefed verbally by the lead researcher and provided with a participant information sheet, translated if needed. They were given at least 2 days to consider their participation. Before undertaking the interview, participants gave formal written consent, using a professional interpreter if required.

**How this fits in**

A number of studies suggest that asylum seekers and refugees (ASR) face barriers accessing primary health care in the UK. However, the policy around charging migrants for health care has recently changed, and no studies have examined ASR access to primary health care since. This study takes a qualitative approach and explores ASR experiences of accessing primary care. The results could be used by healthcare providers to improve access to primary care for ASR.

### Data collection

Semi-structured interviews were used to explore participants’ experiences.^28^ An interview guide was developed collaboratively by the research team, which included an experienced clinician. Interviews were conducted in private at VCO premises, familiar locations for participants. Professional interpreters were offered. Interviews were audiorecorded. Audiorecordings were transcribed by university-approved professional transcription services. Saturation was determined when no new subthemes emerged.^29^^,^^30^

### Ethical considerations

University of Manchester ethical approval was granted. Professionally accredited interpreters were required to sign a confidentiality clause. The nature of the sample meant that many of the participants had experienced traumatic events; therefore, avoiding distress and re-traumatisation were prioritised. The interviewer had >10 years’ experience working with ASR, and gave participants time and the option to withdraw if signs of distress were shown. The data collected were anonymised. Pseudonyms are used in this paper. No identifiable information was collected, and data were stored on password-protected devices. Participants received travel expenses and a £20 voucher.

### Data analysis

Transcripts were read, and experiences of accessing primary care were coded into Penchansky and Thomas’s modified theoretical framework of access; free codes were assigned when this theory was insufficient (Box 2). The six domains were analysed in turn, and subthemes were identified from the coded data. Often, these subthemes traversed multiple main codes; this complexity is addressed in the discussion. Transcripts were analysed individually by two researchers, before undertaking collaborative review to ensure consistency and enhance reliability. The third researcher, having read the transcripts, reviewed the themes and subthemes.

**Box 2.** Subthemes identified from data analysis

**Dimension**	**Subtheme**
Accommodation	Language and interpretation
Awareness	Service navigation and negotiation Knowledge about eligibility for care Knowledge of NHS charging regulations
Affordability	Difficulties paying for medication or prescriptions Difficulties paying for dental treatment
Accessibility	Difficulties paying for transport costs The impact of asylum dispersal accommodation on continuity of care
Availability	Long waiting times for appointments Short appointment times
Acceptability	Positive experiences in medical consultations Negative experiences in medical consultations Discrimination on grounds of race, religion, and immigration status

## Results

In all, 18 participants were interviewed in 2018. The sample included seven males and 11 females aged 18–47 years. Six were refugees, five of whom arrived through family reunion; eight were asylum seekers, and four were refused asylum seekers. Participants had resided in the UK for between 4 months and 5 years. The sample is broadly representative of the current countries of origin of those seeking asylum in the UK^31^ — Pakistan (four), Sudan (four), Syria (four), Iran (two), Libya (one), Eritrea (one), Ivory Coast (one), and Guinea (one).

The dominant narrative expressed by participants was that primary care services were difficult to navigate and negotiate. This was due to multiple factors, many of which demonstrated crossover between the dimensions of access described in Penchansky and Thomas’s modified theory; this interconnectedness is examined in the discussion. For the purposes of analysis, the dominant theme from each dimension is described, with reference to empirical data.

### Accommodation

As in other studies, language barriers were a significant barrier to access.^11^ Many participants struggled to complete GP registration paperwork. None reported being offered linguistic assistance by primary care staff; instead, they capitalised on the skills of informal social contacts:

*‘They gave me a* [registration] *form and I have to fill that form … which is not easy for me. I call one friend … he knows English better than me, and through the phone we filled it. I would send him a picture from the paper, and through the phone he translated to me what I should write here.’*

(Mustafa)

Issues around interpretation were pervasive, and impacted participants’ engagement and treatment. One participant, Amena, was 8 months pregnant and had been referred for investigation of abdominal pain:

Interpreter for Amena: ‘She had a scan on Thursday, but didn’t understand anything. After the scan, she go into the doctor. The doctor herself was very frustrated and upset, because there was no interpreter so she couldn’t communicate with her.’

Researcher: ‘But does she know what to do, say, when she goes into labour? Does she know where she should go to give birth to the baby?’

Interpreter for Amena: ‘No, nothing. She knows nothing.’

Another reported lack of interpretation, resulting in inappropriate dental treatment:

‘I have pain in my teeth too much. I couldn’t speak English very well, and I have nobody to help me, so I am very, very upset. I have pain … and when I see my dentist, they removed my teeth. When I come home, I say my pain is left side and they remove my right side. I go to the dentist and I say: “Where is my tooth?” The dentist says: “Oh, I am so sorry.” They took the wrong one. Still pain is this side … but he’s doing the wrong side, but still pain in my face.’

(Aisha)

Many spoke about more nuanced negative effects of not having an interpreter:

‘The doctor couldn’t speak our language and, at that time, they had no interpreters. It was really bad, yeah. So, we hardly used to go. So, if we had like very pain, then we used to go. Because it was embarrassing, you know, telling doctors in a sign language. It made me feel like I had no self-esteem. I avoided going to the doctors.’

(Laila)

*‘I had to translate a lot for my parents. They didn’t give us any interpreter. I even struggled speaking English with her, but they still didn’t help. I had to go* [to interpret] *during school time. I was in lessons, and I got called that I need to leave high school. It was not a good experience, because I had to leave my study for the doctor.’*

(Sadia)

These accounts highlight the far-reaching physical, mental, and social harm caused by inadequate interpretation — invasive treatment performed incorrectly, individuals feeling embarrassed when accessing care, and children missing school to interpret for parents.

### Awareness

A minority of participants reported receiving information about the NHS on arrival to the UK. When this occurred, it appeared to enhance knowledge of how to access services. However, the majority reported a lack of awareness of NHS structure, and described confusion over how to navigate and negotiate access to health care, including uncertainty about how to arrange appointments. Many were unclear which provider to access for which service:

‘I’d been waiting maybe more than 45 or 50 days after I go to my lawyer. She asked me: “Did you go to the GP?” I said: “I registered, but no one called me.” And then she said she needed to send a mail to my GP, and she gave me a letter. And after the second day they called me, the GP, and they tell me: “Why didn’t you make an appointment?” And I told her: “I don’t know the procedure, you know? How can I know I have to call for an appointment or something?” No one told me about this. Now, I’ve been looking, because I have a problem with my teeth. I don’t know where I need to go — to my GP, near to my place, or if I go to another place?’

(Mustafa)

The confusion between dental services, emergency services, and general practice was prevalent in the narratives. Many participants did not know how to call an ambulance in an emergency, whereas others reported ringing the ambulance service to ‘get advice’:

‘I don’t know … how to use an ambulance service to call them or, like, how to get ambulance.’

(Hassan)

‘I think same like a movie, I need to call 911?’

(Mustafa)

Interviews were carried out 6 months after the introduction of prospective charging for access to community services. None of the participants was aware of the recent change to regulations, and none reported being charged.

### Affordability

GP services in the UK are currently free to ASR and refused asylum seekers.^32^ Asylum seekers are prohibited from working, and receive £37.75 per week. Consequently, they are entitled to free prescriptions, dental treatment, and eye tests under the low-income scheme using an HC2 certificate.^33^ Some participants reported the HC2 application as a lengthy process. Here, the bureaucracy associated with demonstrating low income emerges as a barrier to access:

*‘We received a HC2 letter. We normally get it after* [waiting] *6 months, but if you don’t have that letter … you won’t get your medication. You have to show it.’*

(Laila)

‘With my dad … we went to the GP. He didn’t have that letter, we were waiting for it. But we told them we were asylum seekers, we can’t pay, we can’t afford to buy medicines. The lady said: “You still need an NHS letter. We can’t give you … we can’t just provide you.” We needed the medicine in an emergency, but she didn’t give it us.’

(Sadia)

‘She uses medication, like iron tablets, vitamin D tablets when she came over. So, she went to the GP and explained to them. They say, “We can’t prescribe every single thing you’re asking for.”’

(Interpreter for Sepideh)

Despite being eligible for free dental care, many participants reported being charged:

‘When I went to my dentist, they ask me give £100. They charge me, and that was really a shock, even besides having this, you know, HC2 form. To be honest, very scary when they ask so much money. That was a really bad experience.’

(Bina)

Many of the participants related attending the GP with dental problems. This phenomenon is widespread, due to scarcity of NHS dentists.^34^ It appears especially problematic when combined with lack of awareness and economic challenges.

### Accessibility

Despite participants reporting that medical consultations were provided free of charge, the £37.75 weekly allowance made travel to health centres challenging:

‘It’s not easy, because Home Office … they give you £37.00 every week. So this one, it is for your food … you take some for your food, and you keep for your bus ticket.’

(Mohamed)

‘A ticket would literally cost £5.60, so then we have only £24 left to spend per week, for clothes, to eat, and everything. So that’s why we normally used to walk.’

(Laila)

*‘If I’m not able to afford for a bus fare, even if 1 hour* [away]*, I have to walk rather than take a bus.’*

(Sepideh)

### Availability

Many participants were unhappy with long waiting times to see a GP. Moreover, they felt that GP appointments were too short, and that the biopsychosocial complexity associated with forced migration meant that longer consultations were required:

*‘As a refugee,* [I] *suffer from traumatic events. That’s why* [I] *feel that* [I] *need sometimes more time … in the appointment to explain. And* [I] *feel that* [I] *have psychological problems, and sometimes a problem with* [my] *families and how they react with* [my] *children, so* [I] *need more time to explain.* [I] *feel relieved when they listen and they give time to explain, and then* [I] *do the check. This make* [me] *comfortable and* [I] *can trust this doctor.’*

(Maya)

### Acceptability

Participants’ experiences of medical consultations were mixed. The majority of participants found their interactions with medical professionals to be positive:

‘Well, the doctor in here, they are the most awesome people I have seen in the country.’

(Ali)

‘I have felt very respected and valued. The doctors have been really respecting us.’

(Sadia)

A minority described negative experiences. One participant perceived their GP to be asking inappropriate questions:

*‘I felt … like he needed to finish because he had a meeting. I didn’t feel he cared about what I said, but he just needed to ask … to write. I told him what happened to me* [torture]*, and he asked me a question. I don’t understand why he asked. He said: “Why, if this is in 2013, you are coming now?” This is feeling so bad for me. I think this is not his issue to ask me why. He’s not supposed to ask me this question.’*

(Mustafa)

Several participants reported experiencing discrimination from staff at the surgery owing to their race, religion, or immigration status:

*‘Especially the staff in the reception, the staff working in GP reception, some of them real* [*sic*] *racist. I saw more than three times, and always one girl … she really, really racist. I don’t know how to explain that, but her body language is racist … turned her face other side.* [She] *shouldn’t be racist or make something, because GP for all people. If you want to be racist, be racist in your home or away, not in your job.’*

(Abu)

‘I wear a hijab … but some of them, receptionists, there was that white lady, she was so racist. I was in the queue, it was my turn, but the next lady … there was a girl behind me; she called her first rather than me, even though I was next. I even told her: “I’m next. I’ve been waiting here.” It’s just, it made me feel like, that just because I’m wearing a hijab, just because of my religion, she acted, like, differently with me.’

(Sadia)

‘So, when the receptionist is asking, “are you British, asylum, refugee?” there are people at the back. I might want to keep it confidential, and I don’t want to tell other people. They ask in front of everyone, so you’re embarrassed to tell them: “Yeah, I am.” They just shout, like: “Okay, what’s your status?”’

(Laila)

These excerpts focus on multiple facets, but are united by the description of discrimination. There appears to be a cavalier attitude to confidentiality in some instances.

## DISCUSSION

### Summary

This qualitative study shows that ASR face multiple barriers when accessing primary health care. A lack of awareness of the structure and function of NHS services made the healthcare system difficult to navigate and negotiate. Language barriers and inadequate interpretation in primary care creates both immediate and long-term barriers to accessing care. Discrimination at GP surgeries relating to race, religion, and immigration status was reported. These factors intersect with the difficulties of the asylum system and the multifaceted social marginalisation experienced by ASR in the UK to compound the barriers to accessing health care.

### Strengths and limitations

Participants were known to ASR support organisations, thus the sample cannot be considered representative of all ASR and refused asylum seekers. Moreover, those selecting to take part may be more open or eloquent, or have more germane experiences of healthcare systems. ASR not accessing support organisations may be more marginalised, and thus face greater issues with access.^25^ Language presented some difficulty; despite using trained interpreters, communication was occasionally challenging during the interviews.

The use of Penchansky and Thomas’s modified theoretical framework facilitated a systematic and rigorous analysis of the data. The structure of the theory enhances the replicability of the study, and draws attention to findings that may otherwise have been overlooked. However, at times the theory was not a natural fit, as some subthemes traversed multiple themes. One example is language and interpretation. Although this has been discussed in reference to accommodation, it also impairs access through availability (as the presence of an interpreter effectively shortens consultation time), awareness (as language impairs communication to gain knowledge about services), acceptability (as those with language difficulties face greater discrimination), and affordability (as non-English speakers are less likely to negotiate exemption forms in a timely manner). As a result, the subthemes were not easily compartmentalised; instead, the analysis resulted in an interconnecting web. This brings to the fore the complex and intersecting socioeconomic barriers faced by this population. In light of this, further academic exploration using intersectionality as a theoretical approach may be warranted.^35^^–^37

### Comparison with existing literature

Though this research parallels existing findings showing that ASR face multiple barriers when accessing health care, the data also reveal novel findings. Interestingly, a recent systematic review exploring healthcare professionals’ experiences of providing care for ASR^26^ mirrors the findings in this study of service users. A lack of awareness of the structure and function of NHS services emerged as a dominant theme. Participants told of confusion between GP, dental, and emergency medical provision, and described not knowing that making appointments to see clinicians was a necessary step, and calling 999 for non-urgent medical advice. In some cases, this lack of awareness resulted in a mismatch between expectation and reality, and influenced participants’ perceptions of what was acceptable care. The extent to which lack of awareness features in the data suggests it requires further academic exploration, as well as raising questions about who is responsible for educating new arrivals to the UK about accessing the NHS.

Language barriers and inadequate interpretation in primary care have long been identified,^25^ and are echoed here. Participants’ experiences illustrate the consequences of failing to provide interpretation services, thus attention is drawn to the fundamental importance of effective communication in consultations. Moreover, the pervasive yet nuanced effects of this have been highlighted, including the burden this places on family members, and its impact on patients’ self-esteem.

Participants reported discrimination at GP surgeries relating to their race, religion, and immigration status. Racism has been shown to impair access to health care.^38^ Though institutional racism in the NHS, and concern over the stigma posed by the status of refugee, have been reported in other studies, the authors are unaware of literature directly documenting accounts of discrimination towards patients within UK primary care.^39^^–^45 In the current political climate, with hate crime rising, these accounts of racism and Islamophobia are of concern and warrant further exploration.^46^^,^^47^

It is important to acknowledge the diversity of the study sample and the complexity of the data. Participants had a range of cultural backgrounds, migration trajectories, and health needs. This complexity intersected with the difficulties of the asylum system and the multifaceted social marginalisation experienced by ASR in the UK. For example, the weekly allowance of £37.75 positions ASR in poverty, often unable to buy essential items, including food.^38^ Despite primary health care being free, participants’ accounts demonstrate how poverty can act as a secondary barrier to access. Travel by public transport to appointments is unfeasible due to cost, prolonged waiting for certificates to prove eligibility for free prescriptions left some unable to access medications, and, even with proof of eligibility, dental treatment remained unaffordable. The participants interviewed occupy intersecting positions of social, political, and economic disadvantage, and thus have multiple complex needs that cut across all domains of Penchansky and Thomas’s modified theory.

### Implications for research and practice

The results have drawn attention to the fundamental importance of good basic primary care and the human cost of poor access. Examples of poor practice in all domains of access have been highlighted. In some cases, this contravenes existing guidance. NHS England states that individuals are not required to declare their immigration status on registering with a GP.^48^ The BMA advises that practice staff should not attempt to interpret patients’ immigration status.^49^ Doctors of the World has developed a ‘safe surgeries toolkit’ to help practices facilitate access to primary care services.^50^ Issues warranting further analysis have been identified, including a closer examination of discrimination towards patients in primary care, and deeper exploration of awareness in both ASR patients and healthcare professionals. Additionally, this research supports Saurman’s notion that the dimension of awareness should be included in theoretical thinking on access, particularly when researching marginalised populations whose literacy of mainstream systems may be limited.^21^

It is crucial to highlight examples of good practice in primary care. Many participants reported positive experiences of medical consultations, though they often stated longer appointments were needed to address biopsychosocial complexity. Providing accessible patient-centred care for marginalised populations benefits both patients and the care system long-term, including through the reduction of unscheduled care. The empirical findings have implications for clinical practice. First, provision of good interpretation can have far-reaching biopsychosocial benefits. Second, longer appointment times for those with complex needs establishes an effective therapeutic relationship. Third, recognition of the lack of knowledge some ASR have of the structure and function of the NHS can tackle consequent difficulties for access. Finally, the authors call for action against discrimination from healthcare staff towards patients in primary care. This is a pertinent and timely issue, given the instrumentalisation of the NHS in the government’s current hostile environment policies, and the rise in hate crimes and Islamophobia in the UK.
